# Dosimetric evaluation in Helical TomoTherapy for lung SBRT using Monte Carlo‐based independent dose verification software

**DOI:** 10.1002/acm2.14305

**Published:** 2024-02-18

**Authors:** Takahiro Kosaka, Jun Takatsu, Tatsuya Inoue, Kotaro Iijima, Michimasa Suzuki, Naoya Murakami, Naoto Shikama

**Affiliations:** ^1^ Juntendo University Graduate School of Medicine Bunkyo‐ku Tokyo Japan; ^2^ Department of Radiology Juntendo University Urayasu Hospital Urayasu‐shi Chiba Japan; ^3^ Department of Radiation Oncology Faculty of Medicine Juntendo University Bunkyo‐ku Tokyo Japan; ^4^ Department of Radiation Oncology Graduate School of Medicine Juntendo University Bunkyo‐ku Tokyo Japan

**Keywords:** Helical TomoTherapy, lung cancer, Monte Carlo, secondary independent dose verification, stereotactic body radiation therapy

## Abstract

**Purpose:**

To elucidate the dosimetric errors caused by a model‐based algorithm in lung stereotactic body radiation therapy (SBRT) with Helical TomoTherapy (HT) using Monte Carlo (MC)‐based dose verification software.

**Methods:**

For 38 plans of lung SBRT, the dose calculation accuracy of a treatment planning system (TPS) of HT was compared with the results of DoseCHECK, the commercial MC‐based independent verification software. The following indices were extracted to evaluate the correlation of dosimetric errors: (1) target volume, (2) average computed tomography (CT) value of the planning target volume (PTV) margin, and (3) average CT value of surrounding 2‐mm area of the PTV (PTV ring). Receiver operating characteristic (ROC) analyses determined the threshold for 5% of differences in PTV D_95%_. Then, the 38 plans were classified into two groups using the cutoff values of ROC analysis for these three indices. Dosimetric differences between groups were statistically compared using the Mann–Whitney *U* test.

**Results:**

TPS of HT overestimated by more than 5% in the PTV D_95%_ in 16 of 38 plans. The PTV ring showed the strongest correlation with dosimetric differences. The cutoff value for the target volume, the PTV margin, and the PTV ring was 14.7 cc, −754 HU, and −708 HU, respectively. The area under the curve (AUC) for the target volume, the PTV margin, and the PTV ring were 0.835, 0.878, and 0.932, respectively. Dosimetric errors more than 5% were observed when the PTV volume was less than 15 cc or when the CT value around the target was less than −700 HU.

**Conclusion:**

The TPS of HT might overestimate the PTV dose by more than 5% if any the three indices in this study were below threshold. Therefore, independent verification with an MC‐based algorithm should be strongly recommended for lung SBRT in HT.

## 1 INTRODUCTION

Stereotactic body radiation therapy (SBRT) for early‐stage non‐small cell lung cancer has been reported to have comparable clinical outcomes compared to that of surgery.^1^, ^2^ Continuous developed image‐guided technology and respiratory motion control facilitate to improve accuracy of target localization in lung SBRT.^3^ There are several irradiation methods for lung SBRT, including step‐and‐shoot intensity modulated radiation therapy (IMRT), volumetric modulated arc therapy (VMAT), and Helical TomoTherapy (Accuray, Sunnyvale, CA, USA) (HT). HT has been investigated to improve target coverage while sparing organs at risk (OAR) compared to VMAT and other irradiation techniques.^4^

The latest TomoTherapy unit, Radixact, has equipped with Synchrony system, which enables to track tumors affected by respiratory motion. The Synchrony system in Radixact utilizes a model‐based approach to predict the target position based on the correlation between the internal target position obtained from two‐dimensional kV x‐ray radiographs and the motion of light‐emitting diode markers placed on the patient's chest.^5^ In addition, the treatment time can be shortened due to the high dose rate (1000 MU/min) of Radixact compared with the older units.^6^ Since shorter treatment times can reduce both patient motion and the baseline shift of the target during irradiation,^7^ providing more accurate treatment. Radixact equipped with the Synchrony system has enabled significant improvement in target localization accuracy of lung SBRT using HT.^8^, ^9^ Consequently, Radixact is expected to further expand the indications for lung SBRT in HT.

The treatment planning system (TPS) of HT implements a collapsed cone convolution (CCC) algorithm, which has been reported to overestimate the prescribed dose to a planning target volume (PTV) in lung SBRT.^10^, ^11^, ^12^ The uncertainty associated with heterogeneities correction introduced by the CCC algorithm unavoidably reduced its calculation accuracy. Lung SBRT is particularly sensitive to errors caused by dose calculation algorithms due to the low‐density area surrounding the tumor and the small treatment field. Many studies have also revealed that the dose calculation algorithm affects the accuracy of dose calculations,^4^, ^13^, ^14^, ^15^ and the MC algorithm has been widely considered the gold standard for lung SBRT because of its high calculation accuracy.^12^, ^13^, ^16^ Therefore, the dose calculation with the MC algorithm is pivotal for treatment planning process in lung SBRT with HT.

In conventional measurement‐based quality assurance (QA), homogeneous water‐equivalent phantoms and diode detectors are frequently used. To ensure the deliverability of IMRT plans, pre‐treatment verifications are required.^17^ However, dosimetric errors caused by a model‐based dose calculation algorithm are not able to detect with measurement‐based QA methods, especially in heterogeneous regions such as lung.^18^ The independent verification for pre‐treatment QA using the MC algorithm would be expected to evaluate the accuracy of the model‐based dose calculation algorithm. Moreover, the independent verification software can provide comprehensive plan verifications through a comparison of dose‐volume histograms (DVH), three‐dimensional (3D) gamma analysis.^19^ Consequently, combining secondary dose verification software to measurement‐based QA could improve the sensitivity to detect errors in TPS.^20^ In practice, the software‐based independent verification has been recommended as the dose verification for IMRT in AAPM TG‐219 and previous studies.^21^

Furthermore, numerous studies have investigated various factors such as tumor location, target volume, and heterogeneity of the density around the target can impact on target dose in lung SBRT.^12^, ^22^ The present study evaluated these three factors in lung SBRT with HT using a commercial MC‐based independent verification software, DoseCHECK (Sun Nuclear Corp, Melbourne, FL, USA). Lung SBRT plans were categorized based on tumor location, target volume, and heterogeneity around the target, and dosimetric errors were evaluated for each category. The aim of this study was to quantitatively evaluate the dosimetric errors caused by model‐based algorithms using MC‐based independent validation software.

## 2 METHODS

### 2.1 Patient data and tumor location

This study included 38 lung cancer patients treated with VMAT‐SBRT using Varian TrueBeam (Varian Medical Systems, Inc., Palo Alto, CA) between April 2017 and March 2023. Patient characteristics are shown in Table 1. Four‐dimensional (4D) computed tomography (CT) and free‐breathing CT images were acquired using Aquilion LB (Canon medical systems, Otawara, Japan). In addition, maximum inspiration and expiration breath‐hold CT images were acquired. The image slice thickness was 2‐mm, the matrix size was 512 × 512, and the field of view was 50 cm. This study was approved by our institutional ethics committees.

**TABLE 1:** Patient characteristics.

Characteristic	*n* = 38 (%)
Sex	
Male	30 (78.9)
Female	8 (21.1)
Average age (year)	84
Age (range) (year)	48−94
Target location	
Right upper lobe	11 (28.9)
Right middle lobe	3 (7.9)
Right lower lobe	8 (21.1)
Left upper lobe	10 (26.3)
Left middle lobe	1 (2.6)
Left lower lobe	5 (13.2)

### 2.2 Treatment planning

The gross tumor volume (GTV) was delineated on 4DCT expiratory phase with a CT window level of −500 HU and 1600 HU for the center level and width, respectively. The clinical target volume (CTV) was generated by expanding GTV by 2‐mm so as to include any microscopic tumor extension. Then, PTV was created by adding a 3−5 mm margin to CTV to account for setup uncertainties.^23^, ^24^ IMRT plans for HT were retrospectively generated with Planning Station (Accuray) for TomoTherapy HDA. Other settings were as follows: Jaw width was 2.512 cm, pitch value was 0.143, modulation factor was 2.0, and the calculation grid size was 2 × 2 × 2 mm^3^. The prescribed dose was 42 Gy/4 fractions, which is defined as the dose covering 95% of PTV. The dose constraints of OARs were in accordance with the Japan Clinical Oncology Group (JCOG) 1408 protocol.^25^ Although the TPS for Radixact, Precision (Accuray), was not used in this study, we noted that there was not a clear difference between our Planning Station and Precision with respect to the basic beam model, optimizer, and dose calculation.^6^ Therefore, our findings using the Planning Station can be extended to that are derived from Precision.

### 2.3 Dose verification

**FIGURE 1:**
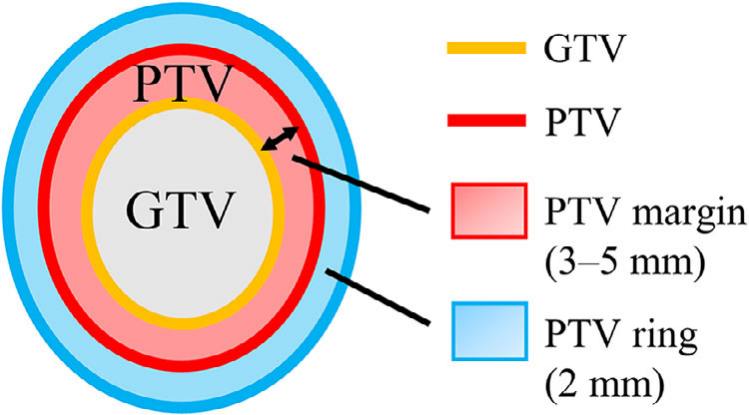
Structure of the quantitative indices used in the present study. GTV, gross tumor volume; PTV, planning target volume.

Independent dose verification of generated plans was performed with DoseCHECK. In the DoseCHECK verification process, the previous study reported that the beam modeling does not need to be changed, only the output parameters need to be adjusted, using the Exradin A1SL ionization chamber (Standard Imaging, Middleton, WI) and the Cheese phantom (Accuray) [Reference: Kosaka et al.]. DoseCHECK output adjustments were performed prior to this study. Dose calculation accuracy of DoseCHECK was previously investigated at our institution, and the results for HT were published by Kosaka et al.^26^ The authors confirmed that the dose calculation accuracy of DoseCHECK was within 1%, except the build‐up region when comparing dosimetric differences with TPS of HT in a homogeneous phantom. DoseCHECK reports statistical uncertainty, which is defined as the standard deviation per voxel. The mean value and standard deviation of the statistical uncertainty for all plans were determined. The dose difference between TPS and DoseCHECK was evaluated by the following formula:

(1) $$\mathrm{Dose}\;\mathrm{Difference}\;=\;100\;\times \;\left({\mathrm{Dose}}_{\mathrm{DC}}-{\mathrm{Dose}}_{\mathrm{TPS}}\right)\;/\;{\mathrm{Dose}}_{\mathrm{TPS}}\left[\%\right]$$

where Dose_DC_ was the dose calculated by DoseCHECK, and Dose_TPS_ was the dose calculated by the TPS. The DVH indices that were compared between TPS and DoseCHECK were PTV D_95%_, PTV D_2%_, and GTV D_98%_. Since the prescribed dose to the target should be delivered within 5% based on the clinical outcome and biological effects,^27^ the criterion for dose differences between TPS and DoseCHECK was chosen to be 5%. Conventional measurement‐based pre‐treatment QA were also performed with ArcCHECK (Sun Nuclear Corp.). Global gamma analyses were performed using a criterion of 3%/2 mm with a 10% threshold.^17^ The gamma criteria were ≤3% for dose and ≤2 mm for distance to agreement (DTA) with a 10% dose threshold. The following indices (shown in Figure 1) were extracted as the factors expected to cause large dosimetric errors in lung SBRT: (1) Target volume, (2) Average CT value of PTV margin (PTV − GTV), and (3) Average CT value of the PTV ring (defined as the 2‐mm thick structure around the PTV).

### 2.4 Statistical analysis

First, since the normal distribution was not confirmed, the correlations between dose differences and indices were evaluated using Spearman's correlation coefficient. Next, ROC analysis was performed for the three indices to determine the threshold for the difference in PTV D_95%_ exceeding 5%. Then, the 38 plans were also classified into two groups using the cutoff values. For each group, the dosimetric differences in PTV D_95%,_ PTV D_2%_, and GTV D_98%_ were calculated and statistically compared using the Mann–Whitney *U* test. Finally, the location of targets was classified into upper lobe or middle/lower lobes. Targets were also classified according to whether the PTV contacted with the chest wall. The Mann–Whitney *U* test was performed on these three indices for patient groups classified by location and isolation of the targets. All analyses were performed using SPSS software (SPSS v. 29.0, SPSS, Inc., Chicago, IL). *p* values < 0.05 were considered statistically significant for all statistical tests. Since all OARs satisfied the dose constraints, no evaluation of OARs was performed in this study.

## 3 RESULTS

### 3.1 Dose differences between TPS and DoseCHECK

**FIGURE 2:**
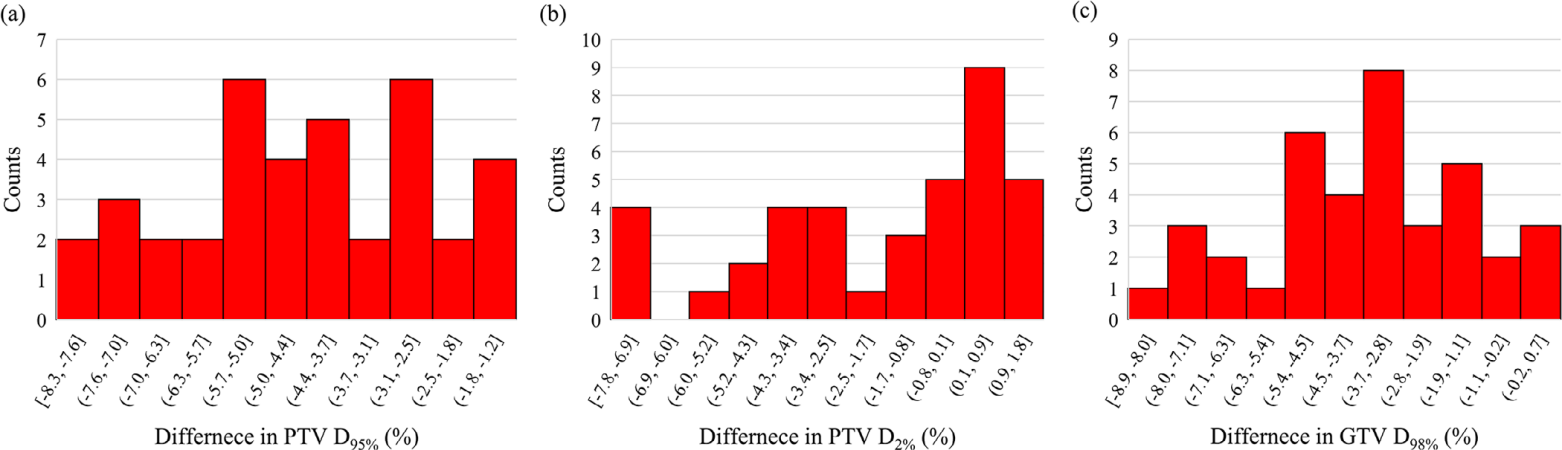
Histograms of dose differences for planning target volume (PTV) D_95%_ (a), PTV D_2%_ (b), and gross tumor volume (GTV) D_98%_ (c).

The median dose differences and range of 38 plans were −3.7% (−8.3–−1.2), −3.1% (−8.9−0.7), and −0.4% (−7.9−1.8) for PTV D_95%_, PTV D_2%,_ and GTV D_98%_, respectively. TPS overestimated PTV D_95%,_ PTV D_2%,_ and GTV D_98%_ by more than 5% in 16 of 38 plans, five of 38 plans, and seven of 38 plans, respectively. The histograms of each dose difference are shown in Figure 2. The average relative statistical uncertainty for lung SBRT plans calculated by DoseCHECK was 0.62% ± 0.23%.

### 3.2 Results obtained from the measurement−based pretreatment QA

The results of the global gamma analyses for these 38 plans were 99.7 ± 0.6%. The lowest gamma passing rate was 97.6%. In this study, the results of the measurement‐based QA on the ArcCHECK satisfied the criteria of AAPM TG‐218 for all lung SBRT plans.

### 3.3 Factors causing dosimetric errors

#### 3.3.1 Correlation coefficients

**FIGURE 3:**
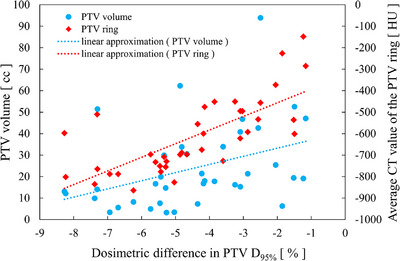
Correlation plots of the dosimetric difference in PTV D_95%_ with PTV volume and average CT value of PTV ring. PTV, planning target volume.

The results of correlation between the three indices are presented in Table 2. Figure 3 shows the scatter plots of the PTVD_95%_ dosimetric difference versus PTV volume and PTV ring. Linear correlations with R2 values between the PTV D_95%_ and the PTV volume, average CT values of the PTV margin, and average CT values of the PTV ring were 0.505, 0.708, and 0.754, respectively.

**TABLE 2:** Spearman's correlation coefficients for the differences in PTV D_95%_, PTV D_2%_, GTV D_98%_.

	PTV volume	PTV margin	PTV ring
PTV D_95%_	0.505^*^	0.708^*^	0.754^*^
PTV D_2%_	0.728^*^	0.543^*^	0.704^*^
GTV D_98%_	0.448^*^	0.678^*^	0.704^*^

*Note*: *, Correlation coefficient was significant at 5% level (two‐tailed).

Abbreviations: GTV, gross tumor volume; PTV, planning target volume.

#### 3.3.2 The result of ROC analysis and dosimetric difference by classification

**FIGURE 4:**
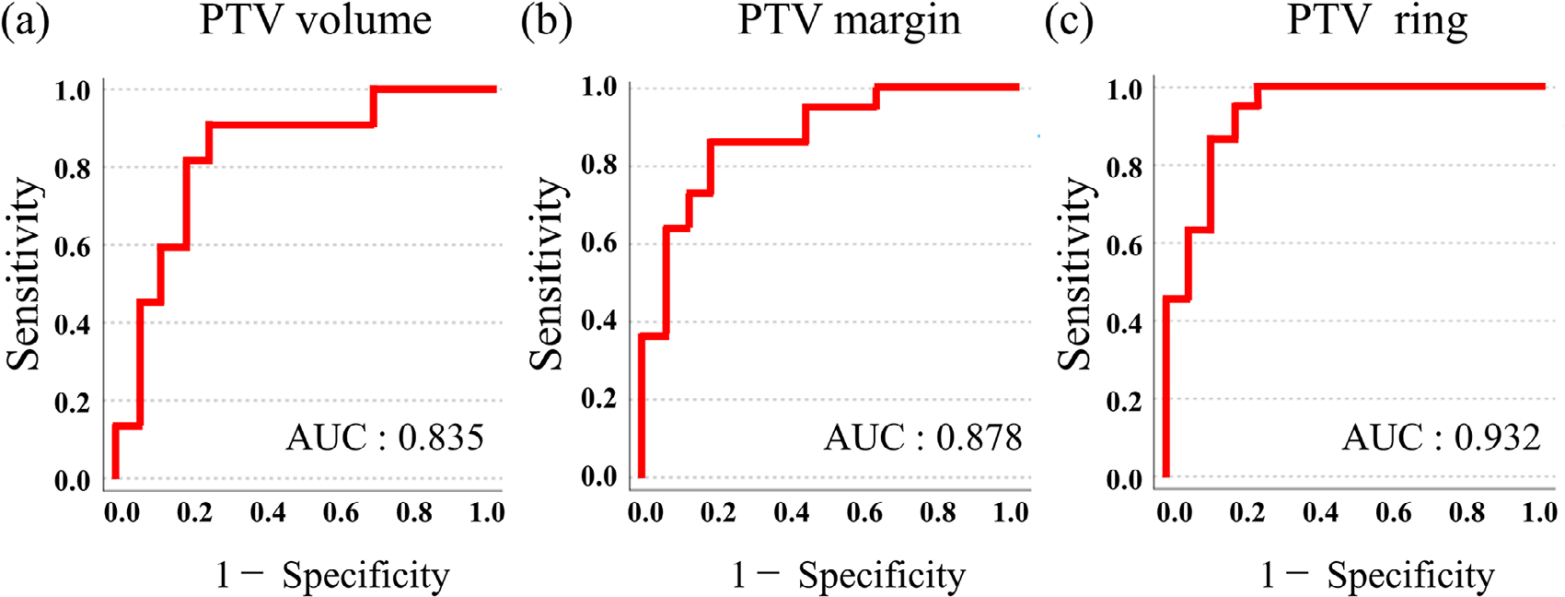
ROC analysis results in the 5% dose difference for each classification. (a) ROC curve of the PTV volume. (b) ROC curve of the CT value of PTV margin. (c) ROC curve of the CT value of PTV ring. AUC, Area under the ROC Curve; PTV, planning target volume; ROC, receiver operating characteristic.

Results of the ROC analyses are shown in Figure 4. The cutoff values for the PTV volume, CT value of PTV margin, and PTV ring were 14.7 cc, −754 HU, and −708 HU, respectively, and the area under the curve (AUC) was 0.835, 0.878, and 0.932, respectively. To simplify the comparison, 38 plans were dichotomized, and the threshold of each index was 15 cc, −700 HU, and −700 HU for the target volume, average CT value of the PTV margin, and average CT value of the PTV ring, respectively. In 14 out of 38 plans, the PTV volume was smaller than 15 cc, the dosimetric errors in PTV D_95%_ exceeded 5% in 12 out of 14 plans. In 20 out of 38 plans the CT value of PTV margin was lower than −700 HU, the dosimetric errors in PTV D_95%_ exceeded 5% in 14 out of 20 plans. In 14 out of 38 plans the CT value of PTV ring was lower than −700 HU, the dosimetric errors in PTV D_95%_ exceeded 5% in 13 out of 14 plans. Table 3 summarizes the median dose differences in PTV D_95%,_ PTV D_2%_, and GTV D_98%_ for each classification. The median dose differences in PTV D_95%_ exceeded 5% for any classification.

**TABLE 3:** Median dose differences for each of the indices examined.

	Median dose difference (Range)		
	PTV D_95%_	PTV D_2%_	GTV D_98%_
All plans (38)	−3.7% (−8.3–−1.2)	−3.1% (−8.9−0.7)	−0.4% (−7.8−1.8)
PTV volume			
< 15 cc (14)	**−6.0**% (−8.3–−1.9)	−4.8% (−8.9–−0.7)	−4.4% (−7.8−0.6)
> 15 cc (24)	−3.4% (−7.3–−1.2)	−2.7% (−7.9−0.7)	0.2% (−7.9−0.7)
*p* value	< 0.05*	< 0.05*	< 0.05*
Average CT value of the PTV margin			
< −700 HU (20)	**−5.4**% (−8.2–−3.1)	−4.5% (−8.9–−2.3)	−3.2% (−7.8−0.7)
> −700 HU (18)	−3.0% (−8.3–−1.2)	−1.6% (−7.7−0.7)	0.4% (−4.1−1.8)
*p* value	< 0.05*	< 0.05*	< 0.05*
Average CT value of the PTV ring			
< −700 HU (14)	**−5.5**% (−8.2–−3.6)	−4.6% (−8.9–−2.3)	−4.1% (−7.8–−0.1)
> −700 HU (24)	−3.2% (−8.3–−1.2)	−2.7% (−7.7−0.7)	0.4% (−4.2−1.8)
*p* value	< 0.05*	< 0.05*	< 0.05*

*Note*: The median dose differences between Dose_DC_ and Dose_TPS_ that were more than 5% is highlighted in bold. * Significant difference between two groups according to the Mann–Whitney *U* test (*p *< 0.05).

Abbreviations: HU, Hounsfield unit; PTV, planning target volume; SD, standard deviation.

#### 3.3.3 Evaluation of the classification according to the target categories

**FIGURE 5:**
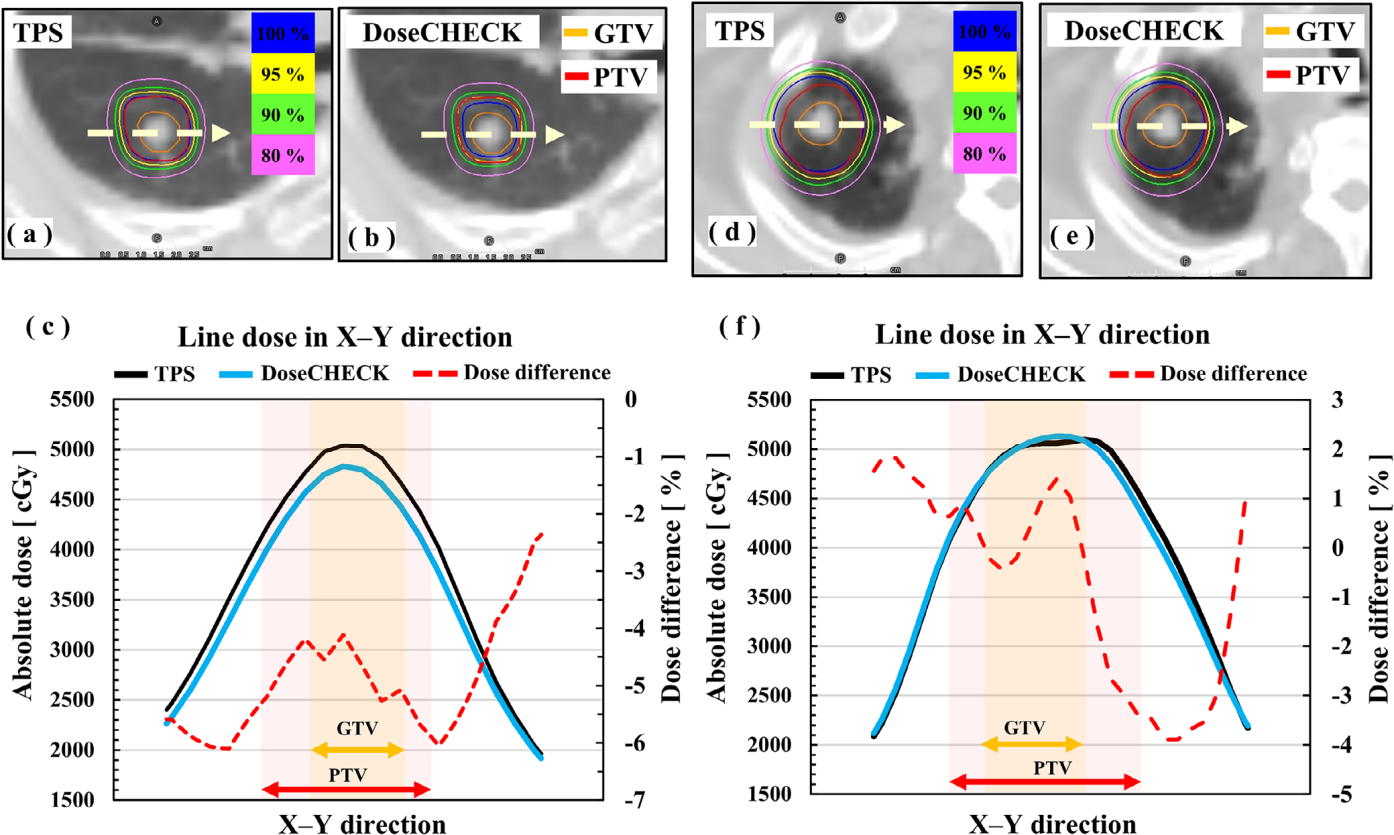
Examples of TPS of HT and DoseCHECK dose distributions and dose profiles according to the patient geometry. (a–c) target of the solitary lesion. (d–f) target of the lesion in contact with the chest wall. GTV, Gross tumor volume; HT, Helical TomoTherapy; PTV, planning target volume; TPS, Treatment planning system.

Table 4 shows the summary of the 38 plans classified by target isolation and location. The median dose difference and range in PTV D_95%_ for the isolated group was −5.3% (−8.3–−1.2). In the case of PTV in contact with the chest wall, the median dose difference and range in PTV D_95%_ was −3.1% (−7.4–−2.5) (*p *< 0.05). In contrast, the target location was not found to affect the statistical dose difference. Figure 5 illustrates the dose distributions and lateral dose profiles for two cases. Figure 5 (a–c) shows the isolated case (PTV located in the right middle lobe, PTV volume = 14.8 cc). The lateral dose profiles revealed that TPS overestimated the dose around PTV by more than 5%. Figure 5 (d–f) shows the case where the target was in contact with the chest wall (PTV located in the right upper lobe, PTV volume = 15.0 cc). The dose difference was +1% on the chest wall and −4% in the lung region.

**TABLE 4:** Summary of the 38 plans classified by target location.

	Non‐contact to the chest wall	Contact to the chest wall	*p* value	Upper lobe	Middle/lower lobe	*p* value
Number of plans	19	19		21	17	
PTV volume (cc)	3.3−93.9	6.3−52.5	0.284	3.5−51.4	3.3−93.9	0.337
Average CT value of PTV margins (HU)	−758	−568	<0.05*	−668	−657	0.601
Average CT value of PTV ring (HU)	−715	−511	<0.05*	−674	−594	0.622
Median dosimetric difference between Dose_DC_ and Dose_TPS_ in PTV D_95%_ (%)	−**5.3** (−8.3–−1.2)	−3.1 (−7.4–−2.5)	<0.05*	−4.8 (−8.3–−1.2)	−4.1 (−6.9–−1.2)	0.170

*Note*: The Median dose differences more than 5% were highlighted in bold. * Significant difference between two groups according to the Mann–Whitney *U* test (*p *< 0.05).

Abbreviations: Dose_DC_, the dose calculated by DoseCHECK; Dose_TPS_, the dose calculated by TPS of Helical TomoTherapy; HU, Hounsfield unit; PTV, planning target volume.

## 4 DISCUSSION

The present study evaluated the dose calculation accuracy of the CCC algorithm of Planning Station using the MC‐based independent verification software for lung SBRT plans. More specifically, we focused on clinical factors associated with dosimetric errors more than 5%. Ono et al. performed the dose comparison between CCC and AcurosXB (Varian Medical Systems, Palo Alto, CA) using 50 lung SBRT plans.^15^ The authors reported that the dose difference in PTV D_95%_ was −3.1 ± 2.0%. Similar result was also reported in the paper published by Sterpin,^28^ in which the authors compared the CCC algorithm of TomoTherapy TPS with a MC method using in‐house software, reporting deviations of up to 4% in PTV D_95%_ of the target for small lung tumors. Their results are generally consistent with the difference found in the present study. Dose differences exceeding this 5% threshold were detected in cases where the target volume was less than 15 cc and the CT value around the PTV was less than −700 HU. In addition, the stronger correlation was observed with the CT density around the PTV than with that of the PTV margin itself. Fourteen out of the 38 plans evaluated revealed the average CT value of −700 HU or less for the PTV ring, and dose differences more than 5% were detected in 13 of them. This result indicated that the physical density of the PTV ring is the most important factor contributing to dosimetric errors of PTV D_95%_. Zheng et al. investigated the target dose differences on various patient factors, such as target size, location, and lung density, between a Pencil Beam and MC calculation algorithms.^29^ The authors concluded that the density around the PTV contributed the most to the dose deterioration of PTV D_95%_. The average CT value outside the PTV could provide the structures around the tumor. For example, if the tumor dose not contact with the chest wall, the surrounding CT values would be lower than that in contact with the chest wall. Aarup et al. evaluated the impact of various lung densities on target dosimetry, revealing the more pronounced disparity in the target dose between AAA and MC algorithms at lower lung densities.^30^ The most important finding of the present study was that dosimetric errors greater than 5% associated with CT values around the PTV less than −700 HU in lung SBRT with HT.

Weyh et al. compared the dose distributions generated by HT with that by VMAT.^4^ They reported that the maximum dose of VMAT plans was on average 7–8% higher than that of HT. This lower maximum dose in HT could potentially contribute to the insufficient dose delivered to the tumor if the CCC algorithm overestimates the dose in low‐density lung regions. In addition, TomoTherapy also requires longer treatment time by 60% than VMAT.^4^, ^31^ Longer treatment time would also increase patient strain and movement during treatment.^7^ Furthermore, the couch in HT does not support pitch and yaw corrections. When considering the actual irregular respiratory movement of the tumor, the dose deterioration in the PTV margin identified in the present study potentially leads to the dose deterioration in the tumor itself. Therefore, in HT, the overestimation of the dose around the PTV caused by the algorithm in lung SBRT needs to be accurately assessed by MC‐based algorithm. Noëlle et al. compared the calculation accuracy between the equivalent path‐length (EPL) algorithm and MC‐algorithm for lung SBRT.^32^ They showed that the clinical implementation of MC‐algorithms could detect the systematic overestimation calculated by the EPL algorithm. In addition, they suggested that the prescription dose for a plan generated by model‐based algorithms should be adjusted, proposing three distinct dose levels depending on the diameter of the tumor (48 Gy for < 3 cm, 51 Gy for 3−5 cm, and 54 Gy for > 5 cm) to prescribe for 95% of PTV based on the MC algorithm. Dose escalation might be promising method to prescribe desired dose to the target for lung SBRT in HT. If the CT value around the PTV was less than −700 HU, then performing Lung SBRT with HT should be required consideration of increasing the prescribed dose. Further studies should be required to determine whether these results could have the impact on the actual tumor control. Savanović et al. if the PTV density is less than 0.36 g/cm^3^, it is recommended that the ITV be overridden to 0.8 g/cm^3^ to improve and simplify treatment planning without affecting treatment safety.^33^ If the independent dose verification with DoseCHECK did not clear the tolerance validation, overriding the density around the tumor might be a solution. However, there is a discrepancy between the isodose line obtained by overwriting and the actual isodose line in the lung.^33^ The verification using MC algorithm actual dose after density override in lung will be subject to future research.

Several present studies have been reported that combining various QA tools improves the sensitivity of error detection.^20^, ^34^, ^35^ O'Daniel et al. assessed the effective risk reduction of combinations of QA methods for IMRT/VMAT treatments.^34^ They showed that the MC‐based secondary dose calculation was available to reduce the overall risk. The utilization of software‐based QA facilitated the identification of dosimetric errors caused by small targets and heterogeneities that were not detected by conventional measurement‐based QA approaches. Therefore, independent verification with DoseCHECK or other Monte Carlo codes should be an important role in lung SBRT with HT. The method of dosimetric evaluation used in the present study can be implemented in any facility where Monte Carlo codes, whether commercial, in‐house, or open source, are available. In addition, the quantitative index obtained in this study could predict the error caused by uncertainty associated with heterogeneities correction. These predictions could help in the evaluation of the patient specific QA for lung SBRT in HT. If any of the three indices were below the thresholds of this study, the results of the TPS calculations should be more carefully verified. One of the limitations of this study was that it did not consider respiratory motion of the target. To assess the dose differences of the target more accurately, it should be desirable to consider actual respiratory motion.

## 5 CONCLUSION

In lung SBRT, the TPS for TomoTherapy was found to cause dosimetric errors more than 5% when the target volume was less than 15cc or the CT value around the target was less than −700 HU. Therefore, we recommend that clinicians should perform dose verification with MC‐based methods when the dosimetric errors was expected to be large.

## AUTHOR CONTRIBUTIONS

Jun Takatsu and Takahiro Kosaka conceived the idea for the study. Tatsuya Inoue, Kotaro Iijima performed literature research, the experiments, and data/statistical analysis. Michimasa Suzuki contributed to the interpretation of the results. Naoya Murakami, Naoto Shikama supervised the work and contributed to the guarantors of integrity of entire study, study concepts/study design, approval of final version of submitted manuscript, and manuscript drafting or manuscript revision for important intellectual content. Takahiro Kosaka prepared the manuscript and Jun Takatsu oversaw the conduct of this study. All authors approved the submitted manuscript and agreed to accept responsibility for any part of this study. The authors would like to thank Toyo Medic Company and Sun Nuclear Corporation for technical assistance.

## CONFLICT OF INTEREST STATEMENT

This study was supported by the research grant from Toyo Medic Corporation, Tokyo, Japan.
